# Knowledge, attitude, performance, and determinant factors of Vitamin D deficiency prevention behaviours among Iranian pregnant women

**DOI:** 10.1186/s13690-021-00712-2

**Published:** 2021-12-10

**Authors:** Farideh Aghaei, Alireza Heidarnia, Hamid Allahverdipour, Mohammad Eslami, Saeideh Ghaffarifar

**Affiliations:** 1 Department of Health Education and Health Promotion, Faculty of Medical Sciences, Tarbiat Modares University, Tehran, Iran; 2 Department of Health Education and Health Promotion, Faculty of Medical Sciences, Tarbiat Modares University, Tehran, Iran; 3 Department of Health Education and Promotion, School of Health, Tabriz, Iran; 4 Research Center of Psychiatry and Behavioral Sciences, Tabriz University of Medical Sciences, Tabriz, Iran; 5 Department of Population Health, Family and Schools Office, Ministry of Health and Medical Education, Tehran, Iran; 6 Medical Education Research Center, Health Management and Safety Promotion Research Institute, Tabriz University of Medical Sciences, Tabriz, Iran

**Keywords:** Vitamin D status, Knowledge, Opinion, Practice, Pregnancy, Predictors

## Abstract

**Background:**

Pregnancy is a high-risk period for vitamin D (Vit D) deficiency, and there is a direct relationship between Vit D deficiency during this period and maternal and fetal complications. Therefore, this study aimed to assess the knowledge, attitude, and practice of pregnant women concerning the adoption of behaviors to prevent Vit D deficiency and identify the determinant factors of such behaviors.

**Methods:**

In this cross-sectional study, 185 pregnant women with a mean age of 27.52 ± 5.9 years were selected from the Health Centers in Tabriz, Iran, using the stratified random sampling between 2018 September 23 and 2019 June 21. Data were collected using a researcher-made questionnaires comprising demographic information, knowledge, attitude, and practice of pregnant women towards Vit D deficiency. The chi-square test and Fisher’s exact test were used to determine the relationship between the demographics of pregnant women and their knowledge and attitude. Moreover, the general linear model test was used to determine the predictors of performance. The *p*-value< 0.05 was considered to be significant in this study.

**Results:**

The findings showed that 85.6% of pregnant women were well aware of the importance and role of Vit D in pregnancy. In addition, 76.7 and 75% of the participants had good knowledge of getting enough Vit D from sunlight and preventing Vit D deficiency in pregnancy, respectively. Moreover, 91.7% of the pregnant women believed that Vit D has a vital role in maternal and fetal health, and 61.1% showed a high level of perceived self-efficiency in preventing Vit D deficiency. In addition, 67.2% of women regarded the unpleasant taste and price of Vit D rich foods, such as seafood, as barriers to get Vit D, and 91.7% mentioned the lack of public places specific to women and living in apartments as barriers to getting enough Vit D from sunlight. According to the results, 57.8 and 79.4% of pregnant women performed at a moderate level in getting Vit D from food and sunlight, respectively. In general, educational attainment (*P*value = 0.02, B = 0.56), pregnancy age (*P*value = 0.04, B = -0.26), parity (*P*value = 0.03, B = -0.45), and perceived self-efficacy of mothers (*P*value < 0.001, B = 0.340) were the determinant factors of getting Vit D from food and sunlight as behaviours to prevent Vit D deficiency in pregnancy.

**Conclusion:**

The findings of the current study revealed that despite the good knowledge of women about the Vit D deficiency during pregnancy, their performance was moderate. The unpleasant taste and high price of seafood were barriers to using them, and the lack of public places specific to women and living in apartments, were barriers to using sunlight. The most important determinant of preventive behaviours was perceived self-efficacy. Developing an awareness program to promote best practices in pregnant women is essential to prevent vitamin D deficiency.

## Background

Pregnancy is a high-risk period for Vit D deficiency [1], which is defined as a serum 25(OH) D level below 20 ng/ml [2]. Pregnancy Vit D deficiency is very prevalent in many parts of the world and can be regarded as a health issue in both developed [3, 4] and developing countries [5–8]. Vit D deficiency is prevalent in Europe and Middle East, despite the high amount of sunlight and UV radiation throughout the year in the Middle East [9–11]. Studies around the world indicate a high prevalence of Vit D deficiency in pregnant women [12–17]. Reported prevalence rates of maternal Vit D deficiency in a systematic review conducted in 2018 were between 70 and 80% across Asia, the Middle East and Africa [18]. Moreover, By a systematic review conducted in 2017 the prevalence of Vit D deficiency in Iranian pregnant women was 56% and mean Vit D concentration have been reported 15.69 ng/ml [19]. Additionally, a recent study showed that 76.8% of the studied Iranian pregnant women were deficient in Vit D [20].

There is a direct relationship between pregnancy Vit D deficiency and maternal and foetal complications [21–23]. Among the maternal complications are increased risk of pregnancy diabetes, bacterial vaginosis, hypertension, preeclampsia, polyhydramnios, and abortion [1, 24, 25]. The foetal complications of Vit D deficiency are low birth weight, premature birth, musculoskeletal abnormalities [24], low levels of serum Vit D, and hypocalcaemia-induced seizures in neonates [1]. As a result, Vit D deficiency prevention can reduce complications during pregnancy and promote maternal and foetal health [26].

Studies have shown that Vit D deficiency is correlated with lifestyle features, such as exposure to sunlight, diet, skin colour, sunscreen use, clothing and coverage, and economic status. Moreover, getting inadequate Vit D from sunlight can be correlated with traditions and religious beliefs. For example, clothing and coverage in some communities, in which most body parts are covered because of traditions or religious beliefs, prevent the exposure of skin to sunlight, as the main source of Vit D intake, and make individuals more prone to Vit D deficiency [24, 27, 28]. The World Health Organization (WHO) has introduced sunlight exposure and Vit D -rich foods as the best sources of Vit D intake during pregnancy [29]. It is obvious that the first step to adopting Vit D deficiency prevention behaviours by pregnant women is the conduction of on-demand educational interventions for enhancing awareness, creating a positive attitude, and adopting Vit D deficiency prevention behaviours [30]. In addition, identifying the relevant behavioural determinant factors can help researchers develop more effective interventions.

Many studies have been conducted to investigate the knowledge, attitude, and performance of people concerning Vit D deficiency [31–36]. Studies conducted on different populations in New Zealand, France, and Saudi Arabia revealed that acquisition of necessary information, nutritional habits, sunlight exposure, and Vit D deficiency prevention behaviours are effective factors in promoting health and eliminating Vit D deficiency [32, 36, 37]. Nevertheless, few similar studies have been conducted specifically on Iranian pregnant women [38]. On the other hand, studies on determinant factors of Vit D deficiency during pregnancy indicate that such factors as educational attainment, income level, Body Mass Index (BMI), regular physical exercise, sunlight exposure, and consumption of Vit D-rich foods and supplements can be Vit D deficiency predictors during pregnancy [17]. To the knowledge of the authors, there are few studies on determinant factors of Vit D deficiency prevention behaviours among Iranian pregnant women. Therefore, this study aimed to determine the knowledge, attitude, and performance of pregnant women concerning the adoption of Vit D deficiency prevention behaviours and also identity the determinant factors of such behaviours among Iranian pregnant women.

## Methods and materials

### Study design and sampling

This cross-sectional study was part of a larger study on the effect of e-learning on knowledge, attitude, and performance of pregnant women concerning Vit D deficiency, which was conducted in Tabriz, Iran, between 2018 September 23 and 2019 June 21. A pilot study was carried out to determine the sample size. The results showed that 30% of pregnant women had acceptable performance concerning Vit D deficiency prevention behaviours. Therefore, the estimated sample size was 165 based on the acceptable performance of pregnant women, results from the pilot study, the estimation error of 7%, and confidence interval of 95% (p = %30, d = %7, CI = %95), using the sample size estimation formula ($n=\frac{Z_{1-\frac{\alpha }{2}}\ P\left(1-P\right)}{d^2}\Big)$. Considering that 10% of questionnaires may be probably incomplete, the sample size increased to 185.

The participants were selected using stratified random sampling. To this end, a list of different regions of Tabriz Municipality and health centres in these regions were prepared. Then, each region was considered a class. Next, the pregnant women were randomly selected based on their population in each region using a software application (Random Number Generator) and then they were invited by telephone contacts. The participants included pregnant women visiting the health centres of Tabriz. The inclusion criteria were living in Tabriz, willingness to participate in the study, educational attainment equivalent to junior high school, non-affliction with medical conditions requiring bed rest, non-affliction with physical and mental disabilities, and non-participation in any Vit D deficiency prevention course in the past.

### Data collection instruments

The data were collected using a researcher-made questionnaire whose face validity was approved qualitatively by interviewing 11 individuals from the target group and quantitatively by calculating the impact score (Impact Score = Frequency (%) × Importance). For qualitative assessment of content validity, the opinions of a panel of 12 experts (two dieticians, one gynaecologist, and nine health education and promotion specialists), were elicited. For the quantitative determination of content validity, the content validity ratio (CVR) and content validity index (CVI) were calculated. Then, the exploratory factor analysis was employed to divide items on knowledge, attitude, and performance into some subgroups and the questionnaire’s items constituted the given subscales. Hereafter, each subscale is called “domain.” Its reliability was evaluated using internal consistency and Cronbach’s alpha. In this regard, Cronbach’s alpha for items related to knowledge, attitude, and performance was 0.65, 0.66, and 0.70, respectively (values larger than 0.7 indicate desired reliability and values larger than 0.6 show moderate reliability). Finally, a questionnaire with the following specifications was designed:

#### Demographic information

The demographic information of the pregnant women included age, educational attainment, job, gestational age, birth order, and residential status. Moreover, three general questions addressed their previous information and status concerning Vit D level as well as the use or non-use of supplements during pregnancy. The participants should respond to these questions by “yes,” “no,” or “don’t know.”

#### Knowledge of pregnant women about Vit D deficiency

This section consisted of 13 items to measure the participants’ knowledge about Vit D in three domains: (1) the importance and role of Vit D in pregnancy, (2) how to get Vit D from sunlight, and (3) how to prevent Vit D deficiency in pregnancy. To estimate the knowledge of the participants, the items were scored between 0 (for the wrong answer) and 1 (for the correct answer). The score of each item was determined by estimating the mean score of correct answers. In the next stage, the score of each domain was determined by calculating the mean score of its items. The total score of knowledge was determined in a range from 0 to 1 by estimating the mean scores on the constituting domains of knowledge. Scores more than 0.66, between 0.33 and 0.66, and lower than 0.33 were regarded as the cutting points representing good, moderate, and poor levels of knowledge, respectively.

#### Attitude of pregnant women towards Vit D deficiency

This section included 12 self-reporting items that evaluate participants’ beliefs about Vit D in four domains: (1) perceptual barriers to consuming Vit D-rich foods, (2) the importance of Vit D in maternal and foetal health, (3) perceived self-efficacy in preventing Vit D deficiency, and (4) perceptual barriers to exposure to sunlight. The items were scored based on a 5-point Likert scale (5: Completely agree, 4: agree, 3: null, 2: disagree, and 1: completely disagree). Six items were scored inversely (A_1,_ A_15_, A_16,_ A_19,_ A_20_, A_21_). Therefore, each item was scored between 1 and 5, and the total score of each domain was obtained from the mean score of its relevant items. The total score of attitude was then obtained in the range of 1–5 by estimating the mean score of its constituent domains. Next, Scores higher than 3.67, between 1.34 and 3.66, and lower than 1.33 were determined as the cutting points representing positive, null, and negative attitudes, respectively.

#### Performance of pregnant women concerning Vit D deficiency

1. This section consisted of seven self-reporting items that evaluate the performance of participants in two domains: (1) consuming Vit D-rich foods, (2) getting Vit D from sunlight. The scoring system was based on the Likert scale anchored with 5 “always,” 4 “often,” 3 “sometimes,” 2 “rarely,” and 1 “never.” Therefore, each item was scored between 1 and 5, and the score of each domain was obtained after calculating the mean score. Then, Scores higher than 3.67, between 1.34 and 3.66, and lower than 1.33 were determined as the cutting points representing good, moderate, and poor performance, respectively.

### Statistical analysis

Data were analysed in SPSS-24. The descriptive tools were used to describe the basic characteristics, knowledge, attitude, and performance of pregnant women. The chi-square test and then the Fisher’s exact test (F-test), if needed, were employed to obtain the relationship of demographics with knowledge, attitude, and performance of pregnant women. The *p*-value< 0.05 was considered to be significant in this study. Moreover, the general linear model test was used to estimate the relationship between two performance domains, namely “consuming Vit D-rich foods” and “getting Vit D from sunlight,” as the dependent variables. In addition, the following potential predictors were investigated: age, educational attainment, job, gestational age, gravida, residential status. The participant’s knowledge in the following domains was investigated: Importance of Vit D in pregnancy, how to get Vit D from sunlight, and how to prevent Vit D deficiency. The participant’s attitude in the following domains was investigated: perceived barriers to consuming Vit D-rich foods, the importance of maternal and foetal health, perceived self-efficacy in Vit D deficiency prevention, and perceived barriers to getting sunlight as the potential determinant factors of adopting Vit D deficiency prevention in pregnant women.

### Ethical considerations

This study was approved by the Ethics Committee of Tarbiat Modares University (Ethical code: IR.MODARES.REC.1397.090). First of all, an informed written consent form was obtained from all participants. The participants were assured that participating in the study was quite voluntary and they could withdraw from the study at any time.

## Results

### General characteristics of participants

In general, 185 pregnant women visiting the health centres of Tabriz were enrolled. Out of the 185 distributed questionnaires, 180 were completed and the five incomplete ones were excluded from the study. Finally, the data obtained from the 180 completed questionnaires were analysed (Response Rate = 97.29%). The majority of the participants were in the age range of 18–35 years (with a mean age of 27.52 ± 5.9 years), housewives (85.0%), living in an apartment (73.9%), nulliparous (61.7%), and in the third trimester of pregnancy (56.7%). Additionally, most of them had a high school diploma (51.7%) and used multivitamin and Vit D supplements (86.7%). The demographic information of the participants is presented in Table 1.

**Table 1:** Baseline characteristics of pregnant women visiting comprehensive health service centers in Tabriz (*n* = 180)

Variable	Number (%)
**Age (Years)**	
< 18	8 (4.4)
18–35	155 (86.1)
35<	17 (9.4)
**Education**	
Less than high school	27 (15.0)
High school	93 (51.7)
Academic degree	60 (33.3)
**Job**	
Unemployed	153 (85.0)
Employed	26 (14.4)
Others^a^	1 (0.6)
**Parity status**	
Nulliparous	111 (61.7)
1	56 (31.1)
2 or more	13 (7.2)
**Gestational trimester**	
1	24 (13.3)
2	54 (30.0)
3	102 (56.7)
**Residential place**	
Apartment	133 (73.9)
House	47 (26.1)
**Intake of vitamin D supplement**	
Yes	156 (86.7)
No	23 (12.8)
I don’t know	1 (0.6)

^a^Students

### Knowledge of pregnant women about Vit D

The results showed that 85.6% of participants were well aware of the importance and role of Vit D in pregnancy, 76.7% of them were well aware of getting Vit D from sunlight exposure, and 75% were aware of how to prevent Vit D deficiency.

The participants’ scores in three domains of knowledge, considering their demographic information, are presented in Table 2. According to this table, among different age ranges, the women aged 18–35 years had the highest level of awareness in three domains of knowledge, namely “importance and role of Vit D in pregnancy,” “how to get Vit D from sunlight,” and “how to prevent Vit D deficiency,” with a mean score of 86.5, 80, and 76.8%, respectively.

**Table 2:** Comparison of pregnant women’s score in knowledge domains based on demographic characteristics (*n* = 180)

				Knowledge domains Score n (%)					
	Importance and role of Vitamin D in Pregnancy			The correct way to use sunlight to get vitamin D			Prevention of Vit D deficiency in Pregnancy		
	Poor (< 0.33)	Moderate (0.33–0.66)	Good (> 0.66)	Poor (< 0.33)	Moderate (0.33–0.66)	Good (> 0.66)	Poor (< 0.33)	Moderate (0.33–0.66)	Good (> 0.66)
**Age (Years)**									
< 18	0(0.0)	2(25.0)	6(75.0)	0(0.0)	4(50.0)	4(50.0)	0(0.0)	5(62.5)	3(37.5)
18–35	0(0.0)	21(13.5)	134(86.5)	0(0.0)	31(20.0)	124(80.0)	0(0.0)	36(23.2)	119(76.8)
35<	0(0.0)	3(17.6)	14(82.4)	0(0.0)	7(41.2)	10(58.8)	0(0.0)	4(23.5)	13(76.5)
^*^P Value	0.500			**0.027**			0.058		
**Education**									
less than high school	0(0.0)	14(51.9)	13(48.1)	0(0.0)	19(70.4)	8(29.6)	0(0.0)	11(40.7)	16(59.3)
high school	0(0.0)	11(11.8)	82(88.2)	0(0.0)	19(20.4)	74(79.6)	0(0.0)	27(29.0)	66(71.0)
Academic degree	0(0.0)	1(1.7)	59(98.3)	0(0.0)	4(6.7)	56(93.3)	0(0.0)	7(11.7)	53(88.3)
*P* Value	**< 0.001**^**a**^			**< 0.001**			**0.006**		
**Job**									
Unemployed	0(0.0)	25(16.3)	128(83.7)	0(0.0)	42(27.5)	111(72.5)	0(0.0)	43(28.1)	110(71.9)
Employed	0(0.0)	0(0.0)	26(100)	0(0.0)	0(0.0)	26(100)	0(0.0)	1(3.8)	25(96.2)
Others	0(0.0)	1(100)	0(0.0)	0(0.0)	0(0.0)	1(100)	0(0.0)	1(100)	0(0.0)
*P* Value	**0.005**			**0.001**			**0.002**		
**Parity status**									
Nulliparous	0(0.0)	14(12.6)	97(87.4)	0(0.0)	23(20.7)	88(79.3)	0(0.0)	32(28.8)	79(71.2)
One child	0(0.0)	8(14.3)	48(85.7)	0(0.0)	12(21.4)	44(78.6)	0(0.0)	10(17.9)	46(82.1)
2 or more Children	0(0.0)	4(30.8)	9(69.2)	0(0.0)	7(53.8)	6(46.2)	0(0.0)	3(23.1)	10(76.9)
*P* Value	0.220			**0.027**			0.296		
**Gestational trimester**									
1	0(0.0)	6(25)	18(75.0)	0(0.0)	5(20.8)	19(79.2)	0(0.0)	4(16.7)	20(83.3)
2	0(0.0)	7(13)	47(87.0)	0(0.0)	8(14.8)	46(85.2)	0(0.0)	18(33.3)	36(66.7)
3	0(0.0)	13(12.7)	89(87.3)	0(0.0)	29(28.4)	73(71.6)	0(0.0)	23(22.5)	79(77.5)
*P* Value	0.310			0.156			0.211		
**Residential place**									
Apartment	0(0.0)	13(9.8)	120(90.2)	0(0.0)	22(16.5)	111(83.5)	0(0.0)	37(27.8)	96(72.2)
House	0(0.0)	13(27.7)	34(72.3)	0(0.0)	20(42.6)	27(57.4)	0(0.0)	8(17.0)	39(83.0)

^*^ Using chi-square test

*P* values < 0.05 are considered statistically significant

Another study result indicated that the pregnant women with an academic degree obtained a higher score on three knowledge domains (98.3, 93.3, and 88.3). Moreover, the results showed the higher score of employed pregnant women on these knowledge domains (100, 100, and 96.2) (Table 2).

### Attitude of pregnant women towards Vit D

The results concerning the four attitude domains indicated that the majority of pregnant women acknowledged the importance of Vit D in maternal and foetal health (91.7%). Moreover, the majority of them had a high level of perceived self-efficacy in Vit D deficiency prevention (61.1%). The findings showed that 5 and 62.2% of the pregnant women, respectively, had a negative and null attitude towards “perceived barriers to the consumption of Vit D-rich foods”. They mentioned the lack of interest in and the high price of seafood as two barriers to consuming Vit D-rich foods. Given the domain of “perceived barriers to getting Vit D from sunlight,” 5 and 86.7% of the pregnant women had a negative and null attitude, respectively. They mentioned the lack of public places specific to women and living in apartments as two barriers to getting Vit D from sunlight. Table 3 compares the scores obtained by the participants on four domains of attitude based on their demographics. Given the “perceived barriers to sunlight exposure,” 16.7% of women in their first trimester had a positive attitude towards their self-efficiency to deal with these barriers; whereas, 3.7 and 8.8% of them in the second and third trimester had a positive attitude towards this domain (Table 3).

**Table 3:** Comparison of pregnant women’s score in attitude domains based on demographic characteristics (*n* = 180)

	Attitude domains Score n (%)											
	Perceptual barriers to the use of foods containing vitamin D			importance of Vitamin D in maternal and fetal health			Perceived self-efficacy in preventing Vit D deficiency			Perceptual barriers to using sunlight		
	Negative (< 1.33)	Neutral (1.34–3.66)	Positive (> 3.67)	Negative (< 1.33)	Neutral (1.34–3.66)	Positive (> 3.67)	Negative (< 1.33)	Neutral (1.34–3.66)	Positive (> 3.67)	Negative (< 1.33)	Neutral (1.34–3.66)	Positive (> 3.67)
**Age (Years)**												
< 18	1(12.5)	6(75)	1(12.5)	0(0.0)	2(25)	6(75)	0(0.0)	3(37.5)	5(62.5)	0(0.0)	8(100)	0(0.0)
18–35	7(4.5)	99(63.9)	49(31.6)	0(0.0)	12(7.7)	143(92.3)	1(0.6)	63(40.6)	91(58.7)	8(5.2)	133(85.8)	14(9)
35<	1(5.9)	7(41.2)	9(52.9)	0(0.0)	1(5.9)	16(94.1)	0(0.0)	3(17.5)	14(82.4)	1(5.9)	15(88.2)	1(5.9)
*P* Value	0.134			0.213			0.292			1.000		
**Education**												
less than high	1(3.7)	23(85.2)	3(11.1)	0(0.0)	4(14.8)	23(85.2)	0(0.0)	11(40.7)	16(59.3)	1(3.7)	23(85.2)	3(11.1)
school high school	4(4.3)	56(60.2)	33(35.5)	0(0.0)	9(9.7)	84(90.3)	0(0.0)	38(40.9)	55(59.1)	3(3.2)	80(86)	10(10.8)
Academic degree	4(6.7)	33(55)	23(38.3)	0(0.0)	2(3.3)	58(96.7)	1(1.7)	20(33.3)	39(65)	5(8.3)	53(88.3)	2(3.3)
*P* Value	0.060			0.152			0.600			0.282		
**Job**												
Unemployed	9(5.9)	95(62.1)	49(32)	0(0.0)	15(9.8)	138(90.2)	1(0.7)	60(39.2)	92(60.1)	6(3.9)	134(87.6)	13(8.5)
employed	0(0.0)	16(61.5)	10(38.5)	0(0.0)	0(0.0)	26(100)	0(0.0)	9(34.6)	17(65.4)	3(11.5)	21(80.8)	2(7.7)
Others	0(0.0)	1(100)	0(0.0)	0(0.0)	0(0.0)	1(100)	0(0.0)	0(0.0)	1(100)	0(0.0)	1(100)	0(0.0)
*P* Value	0.694			0.205			0.828			0.362		
**Parity status**												
Nulliparous	6(5.4)	68(61.3)	37(33.3)	0(0.0)	10(9)	101(91)	1(0.9)	38(34.2)	72(64.9)	3(2.7)	98(88.3)	10(9)
One child	2(3.6)	36(64.3)	18(32.1)	0(0.0)	3(5.4)	53(94.6)	0(0.0)	23(41.1)	33(58.9)	5(8.9)	47(83.9)	4(7.1)
2 or more Children	1(7.7)	8(61.5)	4(30.8)	0(0.0)	2(15.4)	11(84.6)	0(0.0)	8(61.5)	5(38.5)	1(7.7)	11(84.6)	1(7.7)
*P* Value	0.935			0.412			0.292			0.371		
**Gestational trimester**												
1	1(4.2)	18(75)	5(20.8)	0(0.0)	0(0.0)	24(100)	0(0.0)	9(37.5)	15(62.5)	4(16.7)	16(66.7)	4(16.7)
2	3(5.6)	33(61.1)	18(33.3)	0(0.0)	4(7.4)	50(92.6)	1(1.9)	22(40.7)	31(57.4)	1(1.9)	51(94.4)	2(3.7)
3	5(4.9)	61(59.8)	36(35.3)	0(0.0)	11(10.8)	91(89.2)	0(0.0)	38(37.3)	64(62.7)	4(3.9)	89(87.3)	9(8.8)
*P* Value	0.751			0.219			0.670			**0.018**		
**Residential place**												
Apartment	6(4.5)	81(60.9)	46(34.6)	0(0.0)	13(9.8)	120(90.2)	1(0.8)	49(36.8)	83(62.4)	6(4.5)	115(86.5)	12(9)
House	3(6.4)	31(66)	13(27.7)	0(0.0)	2(4.3)	45(95.7)	0(0.0)	20(42.6)	27(57.4)	3(6.4)	41(87.2)	3(6.4)
*P* Value	0.706			0.360			0.705			0.803		

* Using chi-square test

*P* values < 0.05 are considered statistically significant

### Performance of pregnant women concerning Vit D intake

The results showed that the performance of the majority of pregnant women in both domains was at a moderate level, as 57.8 and 79.4% of them, respectively, gained a score of 3.66 and 1.34 (out of 5) in consuming Vit D-rich foods and getting Vit D from sunlight. Table 4 compares the scores obtained by the participants on two performance domains based on their demographics. In “getting Vit D from sunlight,” 19.1% of women living in private houses with a yard and 25.9% of them with educational attainment of lower than a high-school diploma had good performance. In addition, 45% of women with a university degree and 57.7% of employed ones had good performance in this regard (Table 4).

**Table 4:** Comparison of pregnant women’s score in performance domains based on demographic characteristics (*n* = 180)

	Performance domains Score n (%)					
	Eating foods containing vitamin D			Being exposed to sunlight to get vitamin D		
**Age (Years)**	Poor(< 1.33)	Moderate(1.34–3.66)	Good(> 3.67)	Poor(< 1.33)	Moderate(1.34–3.66)	Good(> 3.67)
< 18	0(0.0)	5(62.5)	3(37.5)	1(12.5)	6(75)	1(12.5)
18–35	3(1.9)	90(58.1)	62(40)	6(3.9)	121(78.1)	28(18.1)
35<	0(0.0)	9(52.9)	8(47.1)	0(0.0)	16(94.1)	1(5.9)
*P* Value	0.931			0.365		
**Education**						
less than high	0(0.0)	17(63)	10(37)	0(0.0)	20(74.1)	7(25.9)
school high school	2(2.2)	55(59.1)	36(38.7)	4(4.3)	74(79.6)	15(16.1)
Academic degree	1(1.7)	32(53.3)	27(45)	3(5)	49(81.7)	8(13.3)
*P* Value	0.919			0.576		
**Job**						
Unemployed	3(2)	92(60.1)	58(37.9)	5(3.3)	120(78.4)	28(18.3)
employed	0(0.0)	11(42.3)	15(57.7)	2(7.7)	22(84.6)	2(7.7)
Others	0(0.0)	1(100)	0(0.0)	0(0.0)	1(100)	0(0.0)
*P* Value	0.194			0.348		
**Parity status**						
Nulliparous	3(2.7)	62(55.9)	46(41.4)	7(6.3)	87(78.4)	17(15.3)
One child	0(0.0)	34(60.7)	22(39.3)	0(0.0)	45(80.4)	11(19.6)
2 or more Children	0(0.0)	8(61.5)	5(38.5)	0(0.0)	11((84.6)	2(15.4)
*P* Value	0.815			0.368		
**Gestational trimester**						
1	1(4.2)	14(58.3)	9(37.5)	0(0.0)	20(83.3)	4(16.7)
2	1(1.9)	33(61.1)	20(37)	4(7.4)	40(74.1)	10(18.5)
3	1(1)	57(55.9)	44(43.1)	3(2.9)	83(81.4)	16(15.7)
*P* Value	0.652			0.585		
**Residential place**						
Apartment	2(1.5)	75(56.4)	56(42.1)	7(5.3)	105(78.9)	21(15.8)
House	1(2.1)	29(61.7)	17(36.2)	0(0.0)	38(80.9)	9(19.1)
*P* Value	0.671			0.270		

* Using chi-square test

*P* values < 0.05 are considered statistically significant

### Predictors of Vit D deficiency prevention Behaviours in pregnancy

The results showed that among demographic characteristics, the determinant factors of adoption of Vit D deficiency prevention behaviours by pregnant women, were educational attainment (*P*value = 0.02, B = 0.56) and gestational age (*P*value = 0.04, B = -0.26) in “consuming Vit D-rich foods”, educational attainment (*P*value = 0.02, B = 0.48) and parity (*P*value = 0.03, B = -0.45) in “getting Vit D from sunlight” (Table 5). The results also showed that in attitude domain, the perceived self-efficacy was the determinant factor of pregnant women’s performance in adopting Vit D deficiency prevention behaviours, Respectively (*P*value < 0.001, B = 0.34) in “consuming Vit D-rich foods” and (*P*value < 0.001, B = -0.29) in “getting Vit D from sunlight” (Table 6).

**Table 5:** Model summary and estimation of demographic parameters for predicting the behavior of pregnant women in the prevention of vitamin D deficiency (*n* = 180)

	Practice domains			Practice domains		
	Eating foods containing vitamin D			Being exposed to sunlight to get vitamin D		
	B	(95% CI)	^*^P Value	B	(95% CI)	***P*** Value
**Age (Years)**						
< 18	−0.019	−0.748 to 0.711	0.960	0.135	−0.494 to 0.764	0.672
18–35	−0.036	−0.446 to 0.373	0.861	0.287	−0.066 to 0.640	0.110
> 35 (ref)						
**Education**						
Less than high school	0.562	0.078 to 1.046	**0.023**	0.483	0.066 to 0.900	**0.023**
High school	0.143	−0.163 to 0.449	0.358	− 0.015	− 0.279 to 0.249	0.912
Academic degree (ref)						.
**Job**						
Unemployed	0.964	−0.688 to 2.617	0.251	−0.204	−1.628 to 1.220	0.778
Employed	1.137	−0.565 to 2.839	0.189	−0.474	−1.941 to 0.993	0.525
Others^*^ (ref)						
**Gestational trimester**						
1	−0.171	−0.539 to 0.197	0.360	0.308	−0.009 to 0.625	0.057
2	−0.267	−0.533 to − 0.001	**0.049**	0.102	− 0.127 to 0.331	0.382
3 (ref)						
**Parity status**						
Nulliparous	−0.110	−0.596 to 0.376	0.655	−0.452	−0.870 to − 0.033	**0.035**
1	−0.123	− 0.602 to 0.357	0.614	− 0.194	−0.607 to 0.219	0.355
2 or more (ref)		.	.		.	.
**Residential place**						
Apartment	0.090	−0.203 to 0.382	0.546	0.033	−0.220 to 0.285	0.799
House (ref)		.	.		.	.

^*^Using general linear model test

**Table 6:** Model summary and estimation of model between pregnant women knowledge and attitude domains and behavior of pregnant women in prevention of vitamin D deficiency (*n* = 180)

	Eating foods containing vitamin D			Being exposed to sunlight to get vitamin D		
	B	(95% CI)	*P Value	B	(95% CI)	***P*** Value
**Knowledge domains**						
Importance and role of Vitamin D in Pregnancy	0.349	−0.939 to 1.637	0.593	1.103	−0.007 to 2.212	0.051
The correct way to use sunlight to get vitamin D	1.270	−0.031 to 2.571	0.056	−1.068	−2.190 to 0.053	0.062
Prevention of Vit D deficiency in pregnancy	1.042	−0.035 to 2.118	0.058	0.869	−0.058 to 1.797	0.066
**Attitude domains**						
Perceptual barriers to the use of foods containing vitamin D	0.026	−0.123 to 0.176	0.730	0.078	−0.051 to 0.207	0.235
importance of Vitamin D in maternal and fetal health	−0.052	−0.257 to 0.152	0.614	0.134	−0.043 to 0.310	0.137
Perceived self-efficacy in preventing Vit D deficiency	0.340	0.182 to 0.499	**0.000**	0.298	0.162 to 0.435	**0.000**
Perceptual barriers to using sunlight	0.068	−0.125 to 0.262	0.486	0.065	−0.101 to 0.232	0.439

*Using general linear model test-*P* values less than 0.05 were considered significant

## Discussion

This is among the few studies on the knowledge, awareness, and performance of Iranian pregnant women about Vit D deficiency prevention behaviours. To the knowledge of authors, it is the first study on determinant factors of adopting such behaviours in this group of women.

### Knowledge of pregnant women

This study showed that the majority of the participants had good knowledge of the importance and role of Vit D in pregnancy, and how to get Vit D from sunlight and prevent Vit D deficiency in pregnancy. The results were consistent with the findings of Iniesta et al. who reported good knowledge of English adults on Vit D [39]. Similar results were reported by Toher et al. in Ireland [40], Indicating good knowledge of pregnant women in getting vitamin D from sunlight. But this study reported a low level of knowledge among studied pregnant women about Vit D-rich foods, which is inconsistent with the findings of the present study. This difference in results can be due to the difference in the study time. In other words, technological progress and the popularity of social networks have made it easier for pregnant women to access more relevant information. Other reason behind this discrepancy may be the difference in information provided in countries. For example, the implementation of the Health System Transformation Plan in Iran with a further emphasis on self-care training among different groups may encourage follow-up, thereby enhance the knowledge of pregnant women on health subjects, including the role of vitamins in maternal and fetal health. On the other hand, the assessed knowledge of the participants can depend on the questionnaire’s format. Since the questionnaire employed in this study consisted of multiple-choice items, it could overrate the knowledge level of the participants; whereas, the above-mentioned study used open-response items.

### Attitude of pregnant women

According to the study results, despite the good level of knowledge, the participants did not have a good attitude towards diet and sunlight to prevent Vit D deficiency. They mentioned the high price and unpleasant taste of Vit D-rich foods, such as seafood, as barriers to their consumption. Similar results were obtained from a study on Irish pregnant women [40]. Therefore, the high price of Vit D-rich foods and the lack of interest in their taste were mentioned by the Irish pregnant women as barriers to consuming seafood. Non-consumption of seafood may be rooted in the culture of participants. For example, due to the distance from the sea, seafood is not common in this city. The other reason for the lack of interest in such foods can be the gastrointestinal problems of women during pregnancy.

In this study, the perceived barriers to getting Vit D from sunlight were the lack of public places specific to women and living in apartments. Similar results were reported by al-Jeffry et al. in Saudi Arabia [42]. This similarity can be attributed to cultural similarities between Iran and Saudi Arabia; similar religious beliefs and dressing codes in these two countries result in similar perception of barriers among the female population. A study by Black et al. on the attitude of New Zealand athletes showed that although the majority of the participants tended to be exposed to sunlight, fear of skin cancer was mentioned as a barrier [33]. This difference can be again attributed to cultural and religious differences between Iran and European countries.

According to WHO’s recommendations, sunlight is the major source of Vit D intake to prevent Vit D deficiency among pregnant women [30]. Due to religious beliefs, Iranian women cover most parts of their body in public places, and thus the only chance to get sunlight exposure is in public places specific to women. Moreover, because of living in apartments, there is a little chance of getting sunlight from inside for the majority of them. As a result, the aforementioned factors can act as a barrier to receive sunlight exposure.

### Performance of pregnant women

The results indicated that participants’ performance in consuming Vit D-rich foods was at a moderate level. This is inconsistent with the findings of McAuliffe et al. in Ireland [40]. They stated that even though the Irish women mentioned such barriers as the high price and the lack of interest in seafood, they had good performance in this regard. This difference can be attributed to the difference in dietary habits in the studied areas. As mentioned earlier, because of the distance from the sea, seafood is not popular in this city; whereas seafood is among the popular foods in the city studied by Black et al. (i.e. Dublin), which can justify this difference.

Among other results was the moderate performance of the participants in exposure to sunlight, which is consistent with the findings of Taregh et al. in Pakistan [43]. According to their study, Pakistani women spend less time outdoor and thus are less exposed to sunlight. Pakistan, a neighbouring country to Iran, has religious and cultural similarities with Iran, which can explain these results. A study in China showed that the majority of the participating students were exposed to sunlight 15–20 min per day, and thus had good performance in this regard. The cultural and religious differences between these two countries can justify the difference in the results. Moreover, the physical and psychological conditions of pregnant women can reduce their outdoor activities and thus their exposure to sunlight, as compared to Chinese students. Another reason for the poor performance of Iranian pregnant women was the lack of public places specific to women. Given the dressing codes in Iran, pregnant women cannot get benefit from sunlight exposure. However, presence in public places specific to women can give them the chance to expose some parts of their body, such as hands from fingers to elbows, legs from toes to knees, and neck, to sunlight during physical activities. Another barrier to getting enough Vit D from sunlight among the participants was their place of residence. In this study, the majority of the participants were living in apartments with a balcony without visual privacy, and thus they could not take sunbathe there because of cultural and religious norms.

### Determinant factors of pregnant Women’s behaviour

Although there are many studies on determinant factors of Vit D level, to the knowledge of the authors, it is the first study on the determinant factors of the adoption of Vit D deficiency prevention behaviours by pregnant women. Educational attainment was the most important demographic variable correlated with consuming Vit D-rich foods and Vit D from sunlight. Unexpectedly, women with lower educational attainment had better performance in both cases. As an explanation to this finding, it can be stated that the majority of participants with higher educational attainment were employed and thus predominantly consumed fast foods that contain low vitamin content. Moreover, the best time to get Vit D from sunlight is from 11 am to 3 pm when employed women are working at indoor workplaces. On the other hand, the majority of women with lower educational attainment were housewives and thus they were at home during the given time, allowing them to enjoy Vit D-rich foods and sunlight exposure. Studies conducted by al-Farsi et al. in Saudi Arabia showed similar results on the relationship between educational attainment and serum Vit D level and reported a higher prevalence of Vit D deficiency among women with higher educational attainment [44]. By contrast, Krieger et al. performed a study in Switzerland and showed the positive effects of pregnant women’s educational attainment on their Vit D status [14].

According to the results, the most important determinant factor of getting Vit D from food and sunlight by the participants in the domains of knowledge and attitude was their perceived self-efficacy. In this regard, an increase in the perceived self-efficacy score in Vit D deficiency prevention increased the performance score. Self-efficacy refers to an individual’s confidence in his/her ability to adopt a specific behaviour. Awareness alone is not sufficient for adopting a healthy behaviour; rather, individuals should have self-confidence in this regard. As a result, the first step to adopt a behaviour is perceived self-efficacy. Similar results were reported by Tapia et al. in Thailand [45] and Izadirad et al. [46] in Iran, indicating that perceived self-efficacy in pregnant women can be a predictor of adopting healthy behaviours.

### Strengths and limitations

Random selection of women from all regions of Tabriz with different economic status was a strength of this study. This is because the economic status of the patients may be related to their Vit D intake. The inclusion of both nulliparous and multiparous women was another strength of this study. Since the awareness of nulliparous and multiparous women about correct methods of getting Vit D from sunlight and their performance in this regard may be different, the results can be generalized to both nulliparous and multiparous pregnant women. Although data collection through self-report can be a strength, false-positive responses can turn it into a weakness. Some pregnant women in Iran visit private clinics instead of public health centres. The content and quality of self-nurturing education provided to pregnant women visiting health centres may be different from those visiting private clinics. On the other hand, the economic-cultural status of these two groups can be different. Therefore, the results may not be generalized to all pregnant women in Tabriz. Moreover, the exclusion of rural pregnant women in Tabriz is another limitation of the study. Following up this group of pregnant women and investigating them and their neonates after childbirth can well show the effects of this correct behaviour on maternal and foetal outcomes. Although larger sample sizes may be needed, future studies are recommended to include the women visiting private clinics. Moreover, the measurement of the serum Vit D level of participants can result in more accurate results. In general, Iranian pregnant women have limitations on the use of sunlight, due to the Islamic culture that women should cover their bodies outdoors. In the other hand, women less eat seafood and foods containing vitamin D, due to the high prices. Therefore governmental actions to reduce these restrictions are needed. Creating recreational environments with the possibility of exercising or walking without Islamic clothing for exposing with sunlight and also providing food packages containing vitamin D in health centers, especially for pregnant women with low socio-economic status could be helpful to overcome on Vit D deficiency.

## Conclusion

The majority of the participants had a good level of knowledge on Vit D deficiency prevention during pregnancy. Nevertheless, the unpleasant taste and expensiveness of Vit D-rich foods, such as seafood, were regarded as a barrier to consuming them. In addition, the lack of public places specific to women and living in apartments were also mentioned as barriers to get enough Vit D from sunlight. Despite good knowledge of participants on Vit D deficiency prevention during pregnancy, participants’ performance in such behaviours as consuming Vit D-rich foods and getting Vit D from sunlight, as the main source of this vitamin, was at a moderate level. The most important determinant factor of Vit D deficiency prevention behaviours in pregnant women was perceived self-efficacy. This finding can be taken into account for the development of necessary interventions to enhance the performance of this group in getting adequate Vit D.

## Data Availability

The datasets used and analysed during the current study are available from the corresponding author on reasonable request.

## Abbreviations

Vit D: Vitamin D
